# Seasonal Trends in Emergency Department Visits for Mental and Behavioral Health Conditions Among Children and Adolescents Aged 5–17 Years — United States, January 2018–June 2023

**DOI:** 10.15585/mmwr.mm7238a3

**Published:** 2023-09-22

**Authors:** Lakshmi Radhakrishnan, Kelly Carey, Dylan Pell, Amy Ising, Danielle Brathwaite, Anna Waller, James Gay, Hollie Watson-Smith, Mark Person, Kenan Zamore, Tia Brumsted, Claudia Price, Patti M. Clark, Gabriel Ann Haas, Lauren Gracy, Scott Johnston, Yushiuan Chen, Kyla Muñoz, Meredith Henry, Brittany Willis, Darryl Nevels, Ibitola Asaolu, Sarah Lee, Natalie J. Wilkins, Sarah Bacon, Michael Sheppard, Aaron Kite-Powell, Gary Blau, Michael King, Meghan Whittaker, Rebecca T. Leeb

**Affiliations:** ^1^Detect and Monitor Division, Office of Public Health Data, Surveillance, and Technology, CDC; ^2^Epidemiology and Response Division, New Mexico Department of Health; ^3^Carolina Center for Health Informatics, Department of Emergency Medicine, University of North Carolina at Chapel Hill, Chapel Hill, North Carolina; ^4^Monroe Carell, Jr. Children’s Hospital at Vanderbilt, Nashville, Tennessee; ^5^Community Mental Health & Addiction, Pima County Health Department, Tucson, Arizona; ^6^Center for Policy, Planning and Evaluation, District of Columbia Department of Health, Washington, D.C.; ^7^Department of Behavioral and Community Health, University of Maryland School of Public Health, College Park, Maryland; ^8^District of Columbia Office of the State Superintendent of Education, Washington, D.C.; ^9^Division of Mental Health, Kentucky Department for Behavioral Health, Developmental and Intellectual Disabilities; ^10^Kansas Department of Health and Environment; ^11^Division of Violence Prevention, National Center for Injury Prevention and Control, CDC; ^12^Colorado Department of Public Health and Environment; ^13^Tri-County Health Department, Aurora, Colorado; ^14^Arapahoe County Public Health Department, Greenwood Village, Colorado; ^15^Office of Crisis Services and Suicide Prevention, Tennessee Department of Mental Health and Substance Abuse Services; ^16^Communicable and Environmental Diseases and Emergency Preparedness Division, Tennessee Department of Health; ^17^Division of Family Health and Wellness, Tennessee Department of Health; ^18^Division of Population Health, National Center for Chronic Disease Prevention and Health Promotion, CDC; ^19^Division of Adolescent and School Health, National Center for HIV, Viral Hepatitis, STD, and TB Prevention, CDC; ^20^Office of Strategy and Innovation, National Center for Injury Prevention and Control, CDC; ^21^Substance Abuse and Mental Health Services Administration, Rockville, Maryland; ^22^U.S. Department of Education, Washington, D.C.; ^23^Division of Human Development and Disability, National Center on Birth Defects and Developmental Disabilities, CDC.

**Summary**

**What is already known about this topic?**

Mental and behavioral health conditions are common among school-aged children in the United States.

**What is added by this report?**

Each year, during 2018–2023, among children and adolescents aged 10–17 years, the number and proportion of weekly emergency department visits for eight mental and behavioral health conditions displayed seasonal increases during the fall and spring school semesters relative to the summer period; timing of increases varied by specific condition.

**What are the implications for public health practice?**

Systemic changes that prioritize protective factors, such as physical activity, social support, and inclusive school environments, and incorporate preparedness for increases in mental and behavioral health conditions during back-to-school planning might help improve child and adolescent mental health.

## Abstract

Mental and behavioral health conditions among school-aged children, including substance use disorders and overall emotional well-being, are a public health concern in the United States. Timely data on seasonal patterns in child and adolescent conditions can guide optimal timing of prevention and intervention strategies. CDC examined emergency department (ED) visit data from the National Syndromic Surveillance Program for 25 distinct conditions during January 2018–June 2023 among U.S. children and adolescents aged 5–17 years, stratified by age group. Each year, during 2018–2023, among persons aged 10–14 and 15–17 years, the number and proportion of weekly ED visits for eight conditions increased in the fall school semester and remained elevated throughout the spring semester; ED visits were up to twice as high during school semesters compared with the summer period. Among children aged 5–9 years, the number and proportion of visits increased for five mental and behavioral health conditions. Seasonal increases in ED visits for some conditions among school-aged children warrant enhanced awareness about mental distress symptoms and the challenges and stressors in the school environment. Systemic changes that prioritize protective factors (e.g., physical activity; nutrition; sleep; social, community, or faith-based support; and inclusive school and community environments) and incorporate preparedness for increases in conditions during back-to-school planning might improve child and adolescent mental health.

## Introduction

Mental and behavioral health conditions among school-aged children, including substance use disorders and overall emotional well-being, are a public health concern in the United States (*1**–**3*). School, particularly the beginning of a new school year, can be both exciting and increase worries and stress for children and adolescents.* School staff members might also recognize exacerbations of these conditions. Timely data on seasonal patterns in child and adolescent conditions can help guide the optimal timing of prevention and intervention strategies to promote child and adolescent long-term well-being.

## Methods

CDC examined emergency department (ED) visit data from the National Syndromic Surveillance Program (NSSP) during January 2018–June 2023 to calculate changes in the number and proportion of ED visits for mental and behavioral health conditions among children and adolescents aged 5–17 years; visits from 1,919 facilities in 46 states were included.^†^ Predetermined *International Classification of Disease, Tenth Revision, Clinical Modification* diagnostic categories from the Healthcare Cost and Utilization Project (HCUP) Clinical Classifications Software Refined^§^ (version 2022; HCUP) tool were used; categories included were initially limited to those corresponding to 27 distinct conditions using a one-to-many approach (Supplementary Box, https://stacks.cdc.gov/view/cdc/131758). Among these categories, eight (30%) had enough data for reliable visit estimates (relative SE <30%) for all age groups and were retained in the final analysis. Results were reported on categories with consistent and significant increases during the study years.

Surveillance periods were designated as the fall school semester (calendar weeks 37–53; September–December) and spring school semester (calendar weeks 1–23; January–June). Each was compared with the immediately preceding summer period (calendar weeks 24–36; June–September) (Supplementary Table, https://stacks.cdc.gov/view/cdc/132871) (*1*). ED visit ratios and 95% CIs were used to measure relative change in the proportion of visits. Visit ratio was defined as the proportion of all ED visits for a selected mental and behavioral health condition during the school semester (fall or spring) divided by the proportion of ED visits for that condition during the immediately preceding summer period. Ratios >1 indicated a higher proportion of ED visits with the condition during the surveillance period than during the comparison period; ratios <1 indicated a lower proportion of ED visits with the condition during the comparison period than during the surveillance period. CIs that excluded 1 were considered statistically significant. Absolute differences and percent changes were used to measure the difference in mean weekly ED visit numbers during the school semester (fall or spring) compared with the preceding summer period. Results were stratified by age group: 5–9, 10–14, and 15–17 years. This activity was reviewed by CDC, deemed not research, and was conducted consistent with applicable federal law and CDC policy.^¶^

## Results

Each year, during 2018–2023, among persons aged 10–14 and 15–17 years, the number and proportion of weekly ED visits displayed seasonal patterns for depressive disorders, suicidal ideation or self-harm, trauma- and stressor-related disorders, cannabis-related disorders, lifestyle or life management factors, mood disorders, poisoning by drugs, and symptoms of mental and substance use conditions. Compared with the summer period, higher mean weekly visit numbers and relative proportion of visits were observed during the fall school semester (i.e., depressive disorders, suicidal ideation or self-harm, and trauma- and stressor-related disorders) and spring school semester (i.e., depressive disorders, suicidal ideation or self-harm, trauma- and stressor-related disorders, lifestyle or life management factors, mood disorders, poisoning by drugs, and symptoms of mental and substance use conditions). ED visits briefly decreased each year corresponding to the typical winter holiday break period during the last week of November and December, followed by a return to previous levels (Figure) (Supplementary Figure, https://stacks.cdc.gov/view/cdc/132872).

**FIGURE:**
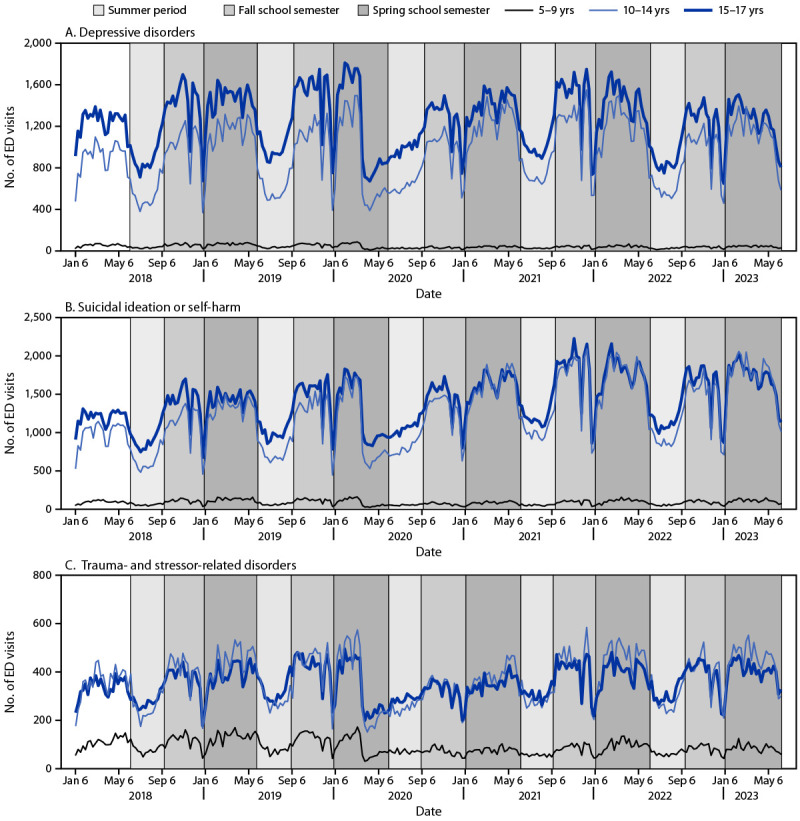
Weekly trends in the number of emergency department visits* for depressive disorders (A), suicidal ideation or self-harm (B), and trauma- and stressor-related disorders (C) among children and adolescents aged 5–17 years, by age group — National Syndromic Surveillance Program,† United States, January 2018–June 2023^§^ **Abbreviation:** ED = emergency department. * To reduce artifactual impact from changes in reporting patterns, analyses were restricted to facilities with a coefficient of variation for ED visits ≤40 and average weekly informative discharge diagnosis ≥75% complete with consistent discharge diagnosis code formatting during January 2018–June 2023. ^†^ National Syndromic Surveillance Program is a collaboration among CDC; local and state health departments; and federal, academic, and private sector partners. https://www.cdc.gov/nssp/index.html ^§^ Summer period (calendar weeks 24–36; June–September); fall school semester (calendar weeks 37–53; September–December); spring school semester (calendar weeks 1–23; January–June).

Among persons aged 10–14 years and 15–17 years, the proportion of ED visits for depressive disorders increased in both the fall and spring school semesters each year during 2018–2023 compared with the preceding summer period (range of visit ratios across fall and spring school semesters: 1.19–1.95 among persons aged 10–14 years and 1.16–1.60 among those aged 15–17 years), suicidal ideation or self-harm (1.13–2.00 and 1.15–1.74, respectively) and trauma- and stressor-related disorders (1.07–1.62 and 1.05–1.43, respectively). During the spring school semester, the proportion of visits increased for four additional conditions: lifestyle or life management factors (range of visit ratios for spring school semesters: 1.32–1.88 among persons aged 10–14 years and 1.07–1.64 among those aged 15–17 years, respectively), mood disorders (1.12–1.73 and 1.13–1.56, respectively), poisoning by drugs (1.05–2.03 and 1.10–1.84, respectively), and symptoms of mental and substance use conditions (1.19–1.47 and 1.08–1.55, respectively) when compared with the preceding summer period (Table 1). For cannabis-related disorders, the proportion of ED visits increased among both children and adolescents aged 10–14 years and 15–17 years during fall 2018 (visit ratio: 1.25 among persons aged 10–14 years and 1.13 among those aged 15–17 years, respectively), spring 2019 (1.36 and 1.22, respectively), spring 2020 (1.61 and 1.66, respectively), fall 2021 (1.39 and 1.17, respectively), spring 2022 (1.91 and 1.48, respectively), and spring 2023 (1.62 and 1.24, respectively). The proportion of weekly ED visits increased among children aged 5–9 years for depressive disorders, suicidal ideation or self-harm, trauma- and stressor-related disorders, mood disorders, and symptoms of mental and substance use conditions.

**TABLE 1:** Emergency department visit ratios*^,^^†^ for mental and behavioral health conditions among children and adolescents aged 5–17 years, by age group — National Syndromic Surveillance Program,^§^ United States, January 2018–June 2023

Mental and behavioral health condition/Age group, yrs	Period	Visit ratio (95% CI)					
		2018–2019	2019–2020	2020–2021	2021–2022		2022–2023
**Depressive disorders**							
5–9	Fall	1.66 (1.47–1.87)	1.45 (1.29–1.63)	1.27 (1.10–1.47)	1.44 (1.25–1.65)		0.99 (0.86–1.14)
	Spring	1.59 (1.40–1.79)	1.57 (1.37–1.81)	1.23 (1.07–1.41)	1.38 (1.19–1.58)		1.31 (1.13–1.51)
10–14	Fall	1.63 (1.58–1.67)	1.46 (1.42–1.50)	1.47 (1.43–1.51)	1.42 (1.39–1.46)		1.19 (1.16–1.22)
	Spring	1.57 (1.53–1.62)	1.95 (1.89–2.01)	1.46 (1.42–1.50)	1.32 (1.29–1.36)		1.38 (1.34–1.42)
15–17	Fall	1.40 (1.37–1.43)	1.31 (1.28–1.34)	1.25 (1.22–1.27)	1.33 (1.31–1.36)		1.16 (1.14–1.19)
	Spring	1.35 (1.32–1.38)	1.60 (1.56–1.63)	1.20 (1.17–1.23)	1.29 (1.27–1.32)		1.21 (1.18–1.24)
**Suicidal ideation or self-harm**							
5–9	Fall	1.62 (1.48–1.77)	1.48 (1.36–1.61)	1.27 (1.15–1.40)	1.64 (1.49–1.80)	1.07 (0.98–1.17)	
	Spring	1.64 (1.50–1.79)	1.56 (1.41–1.73)	1.20 (1.09–1.32)	1.71 (1.56–1.88)	1.39 (1.27–1.52)	
10–14	Fall	1.52 (1.48–1.56)	1.41 (1.38–1.44)	1.52 (1.48–1.55)	1.45 (1.42–1.48)	1.13 (1.11–1.15)	
	Spring	1.50 (1.46–1.54)	2.00 (1.94–2.05)	1.51 (1.48–1.55)	1.38 (1.35–1.41)	1.38 (1.35–1.41)	
15–17	Fall	1.37 (1.34–1.40)	1.28 (1.26–1.31)	1.32 (1.30–1.35)	1.36 (1.34–1.39)	1.15 (1.12–1.17)	
	Spring	1.31 (1.28–1.34)	1.74 (1.70–1.78)	1.30 (1.28–1.33)	1.34 (1.31–1.37)	1.25 (1.22–1.28)	
**Trauma- and stressor-related disorders**							
5–9	Fall	1.31 (1.21–1.42)	1.29 (1.19–1.39)	1.04 (0.95–1.13)	1.35 (1.23–1.47)		0.87 (0.80–0.95)
	Spring	1.30 (1.20–1.40)	1.64 (1.50–1.79)	0.82 (0.75–0.89)	1.43 (1.31–1.57)		1.00 (0.91–1.09)
10–14	Fall	1.36 (1.31–1.42)	1.25 (1.20–1.30)	1.24 (1.18–1.29)	1.36 (1.31–1.42)		1.07 (1.03–1.11)
	Spring	1.35 (1.29–1.41)	1.62 (1.55–1.70)	1.18 (1.13–1.23)	1.38 (1.33–1.44)		1.27 (1.22–1.33)
15–17	Fall	1.20 (1.15–1.25)	1.13 (1.09–1.18)	1.08 (1.04–1.13)	1.21 (1.16–1.26)		1.05 (1.01–1.09)
	Spring	1.24 (1.19–1.30)	1.43 (1.37–1.50)	1.06 (1.01–1.10)	1.23 (1.18–1.28)		1.15 (1.11–1.20)
**Cannabis-related disorders**							
5–9	Fall	1.23 (0.73–2.07)	0.95 (0.64–1.39)	0.98 (0.76–1.27)	0.47 (0.37–0.60)		0.63 (0.52–0.77)
	Spring	1.28 (0.77–2.15)	2.94 (2.02–4.27)	1.27 (1.00–1.61)	0.85 (0.69–1.05)		1.04 (0.86–1.26)
10–14	Fall	1.25 (1.14–1.38)	1.07 (0.98–1.16)	0.99 (0.91–1.07)	1.39 (1.28–1.50)		1.13 (1.06–1.21)
	Spring	1.36 (1.24–1.49)	1.61 (1.47–1.76)	1.08 (0.99–1.17)	1.91 (1.77–2.05)		1.62 (1.52–1.73)
15–17	Fall	1.13 (1.09–1.18)	1.06 (1.02–1.10)	0.98 (0.94–1.02)	1.17 (1.12–1.21)		0.99 (0.96–1.03)
	Spring	1.22 (1.17–1.27)	1.66 (1.59–1.73)	0.98 (0.94–1.02)	1.48 (1.42–1.54)		1.24 (1.19–1.28)
**Lifestyle or life management factors**							
5–9	Fall	1.29 (1.00–1.66)	1.17 (0.91–1.50)	0.87 (0.66–1.16)	0.94 (0.72–1.23)		0.92 (0.71–1.21)
	Spring	1.04 (0.80–1.36)	1.32 (0.98–1.78)	0.86 (0.65–1.13)	1.08 (0.83–1.41)		1.56 (1.21–2.02)
10–14	Fall	1.44 (1.33–1.56)	1.25 (1.17–1.35)	1.28 (1.19–1.37)	1.30 (1.22–1.39)		0.98 (0.92–1.05)
	Spring	1.39 (1.28–1.50)	1.88 (1.74–2.04)	1.39 (1.30–1.49)	1.32 (1.24–1.41)		1.41 (1.32–1.51)
15–17	Fall	1.08 (1.02–1.15)	1.05 (1.00–1.11)	1.08 (1.03–1.14)	1.15 (1.09–1.21)		0.93 (0.88–0.98)
	Spring	1.07 (1.01–1.14)	1.64 (1.54–1.74)	1.11 (1.05–1.18)	1.19 (1.12–1.25)		1.16 (1.10–1.23)
**Mood disorders**							
5–9	Fall	1.62 (1.45–1.81)	1.31 (1.18–1.45)	1.24 (1.09–1.40)	1.32 (1.17–1.48)		0.81 (0.72–0.91)
	Spring	1.47 (1.31–1.65)	1.41 (1.24–1.60)	1.12 (0.99–1.26)	1.27 (1.13–1.43)		1.08 (0.96–1.21)
10–14	Fall	1.31 (1.24–1.40)	1.28 (1.21–1.35)	1.12 (1.06–1.18)	1.24 (1.18–1.31)		0.96 (0.92–1.01)
	Spring	1.32 (1.24–1.40)	1.73 (1.62–1.84)	1.12 (1.05–1.18)	1.32 (1.25–1.39)		1.22 (1.16–1.28)
15–17	Fall	1.17 (1.09–1.25)	1.08 (1.01–1.15)	1.08 (1.01–1.15)	1.26 (1.19–1.33)		1.01 (0.95–1.07)
	Spring	1.18 (1.10–1.26)	1.56 (1.46–1.68)	1.13 (1.06–1.20)	1.37 (1.29–1.45)		1.20 (1.13–1.27)
**Poisoning by drugs, initial encounter**							
5–9	Fall	0.76 (0.69–0.84)	0.76 (0.69–0.84)	0.86 (0.78–0.96)	0.72 (0.66–0.80)		0.61 (0.56–0.67)
	Spring	0.73 (0.66–0.81)	1.33 (1.20–1.49)	0.76 (0.69–0.84)	0.83 (0.76–0.91)		0.82 (0.75–0.90)
10–14	Fall	1.20 (1.14–1.26)	1.04 (0.99–1.09)	1.37 (1.31–1.43)	1.14 (1.10–1.19)		0.90 (0.86–0.93)
	Spring	1.18 (1.11–1.24)	2.03 (1.93–2.14)	1.34 (1.29–1.40)	1.05 (1.00–1.09)		1.08 (1.03–1.13)
15–17	Fall	1.22 (1.18–1.27)	1.11 (1.07–1.15)	1.20 (1.16–1.24)	1.20 (1.16–1.24)		1.01 (0.97–1.04)
	Spring	1.15 (1.11–1.20)	1.84 (1.76–1.91)	1.16 (1.12–1.21)	1.18 (1.14–1.22)		1.10 (1.06–1.14)
**Symptoms of mental and substance use conditions**							
5–9	Fall	1.42 (1.35–1.50)	1.23 (1.18–1.29)	1.29 (1.22–1.37)	1.20 (1.14–1.27)		0.84 (0.80–0.88)
	Spring	1.42 (1.35–1.50)	1.32 (1.24–1.40)	1.12 (1.06–1.19)	1.25 (1.18–1.31)		1.07 (1.01–1.12)
10–14	Fall	1.39 (1.34–1.44)	1.16 (1.12–1.20)	1.29 (1.25–1.34)	1.23 (1.19–1.27)		0.97 (0.95–1.00)
	Spring	1.43 (1.38–1.49)	1.47 (1.42–1.53)	1.27 (1.23–1.31)	1.34 (1.30–1.38)		1.19 (1.16–1.23)
15–17	Fall	1.22 (1.17–1.27)	1.05 (1.01–1.09)	1.11 (1.07–1.15)	1.16 (1.12–1.20)		0.99 (0.96–1.02)
	Spring	1.30 (1.25–1.35)	1.55 (1.49–1.61)	1.08 (1.04–1.12)	1.33 (1.28–1.37)		1.19 (1.15–1.23)

**Abbreviation:** ED = emergency department.

* Visit ratio was defined as the proportion of all ED visits for a selected mental and behavioral health condition during the school semester (fall school semester [calendar weeks 37–53, September–December]; spring school semester [calendar weeks 1–23, January–June]) divided by the proportion of ED visits for that condition during the immediately preceding summer period (calendar weeks 24–36, June–September).

^†^ To reduce artifactual impact from changes in reporting patterns, analyses were restricted to facilities with a coefficient of variation of ED visits ≤40 and average weekly informative discharge diagnosis ≥75% complete with consistent discharge diagnosis code formatting during January 2018–June 2023.

^§^ National Syndromic Surveillance Program is a collaboration among CDC; local and state health departments; and federal, academic, and private sector partners. https://www.cdc.gov/nssp/index.html

The number of weekly visits also increased for all eight mental and behavioral health conditions in the fall and spring semesters when compared with the preceding summer period among children and adolescents aged 10–14 and 15–17 years, except when comparing the spring 2020 school semester with the preceding summer 2019 period; cannabis-related disorders were the only exception in which negative percent change (−4.6%) in weekly visits was also observed among adolescents aged 15–17 years during fall 2020 (Table 2). Weekly ED visits among children aged 5–9 years were higher during school semesters when compared with corresponding summer periods for depressive disorders, suicidal ideation or self-harm, trauma- and stressor-related disorders, mood disorders, and symptoms of mental and substance use conditions, except during the spring 2020 school semester; the volume of visits was low for conditions examined when compared with children and adolescents aged 5–17 years.

**TABLE 2:** Total and weekly emergency department visits* and percentage change^†^ from the fall and spring school semester compared with the summer period for mental and behavioral health conditions among children and adolescents aged 5–17 years, by year and age group — National Syndromic Surveillance Program,^§^ United States, January 2018–June 2023

Mental and behavioral health condition/School year	Age group, yrs	Mean weekly no. of visits			Change in mean weekly no. of visits (% Change)	
		Summer	Fall	Spring	Fall	Spring
**Depressive disorders**						
2018–2019	5–9	28.5	55.6	62.5	27.1 (95.1)	34.0 (119.3)
	10–14	528.7	1,013.1	1,132.6	484.4 (91.6)	603.9 (114.2)
	15–17	904.3	1,413.4	1,434.1	509.1 (56.3)	529.8 (58.6)
2019–2020	5–9	31.4	58.0	27.2	26.6 (84.7)	−4.2 (−13.4)
	10–14	591.5	1,072.9	638.6	481.4 (81.4)	47.1 (8.0)
	15–17	1,003.8	1,516.3	931.6	512.5 (51.1)	−72.2 (−7.2)
2020–2021	5–9	23.2	28.4	38.5	5.2 (22.4)	15.3 (65.9)
	10–14	645.2	972.4	1,238.1	327.2 (50.7)	592.9 (91.9)
	15–17	1,006.9	1,263.9	1,413.4	257.0 (25.5)	406.5 (40.4)
2021–2022	5–9	22.8	37.1	40.6	14.3 (62.7)	17.8 (78.1)
	10–14	748.9	1,194.5	1,167.4	445.6 (59.5)	418.5 (55.9)
	15–17	1,017.8	1,458.1	1,367.1	440.3 (43.3)	349.3 (34.3)
2022–2023	5–9	22.2	33.1	38.6	10.9 (49.1)	16.4 (73.9)
	10–14	617.7	986.5	1,065.8	368.8 (59.7)	448.1 (72.5)
	15–17	886.5	1,219.7	1,180.9	333.2 (37.6)	294.4 (33.2)
**Suicidal ideation or self-harm**						
2018–2019	5–9	52.9	100.7	119.7	47.8 (90.4)	66.8 (126.3)
	10–14	628.2	1,126.2	1,282.5	498.0 (79.3)	654.3 (104.2)
	15–17	913.2	1,394.1	1,404.4	480.9 (52.7)	491.2 (53.8)
2019–2020	5–9	59.2	111.7	51.0	52.5 (88.7)	−8.2 (−13.9)
	10–14	727.5	1,271.0	804.2	543.5 (74.7)	76.7 (10.5)
	15–17	1,016.1	1,504.5	1,029.5	488.4 (48.1)	13.4 (1.3)
2020–2021	5–9	49.9	61.0	80.7	11.1 (22.2)	30.8 (61.7)
	10–14	817.9	1,275.1	1,626.8	457.2 (55.9)	808.9 (98.9)
	15–17	1,075.3	1,431.9	1640.0	356.6 (33.2)	564.7 (52.5)
2021–2022	5–9	47.7	88.8	105.9	41.1 (86.2)	58.2 (122.0)
	10–14	1,037.5	1,689.6	1,685.6	652.1 (62.9)	648.1 (62.5)
	15–17	1,227.7	1,795.4	1,708.6	567.7 (46.2)	480.9 (39.2)
2022–2023	5–9	54.5	87.7	101.3	33.2 (60.9)	46.8 (85.9)
	10–14	956.8	1,455.4	1,648.8	498.6 (52.1)	692.0 (72.3)
	15–17	1,177.5	1,594.3	1,619.4	416.8 (35.4)	441.9 (37.5)
**Trauma- and stressor-related disorders**						
2018–2019	5–9	75.8	116.6	136.1	40.8 (53.8)	60.3 (79.6)
	10–14	249.5	400.5	458.9	151.0 (60.5)	209.4 (83.9)
	15–17	278.8	373.9	407.8	95.1 (34.1)	129.0 (46.3)
2019–2020	5–9	76.8	125.9	69.4	49.1 (63.9)	−7.4 (−9.6)
	10–14	280.0	435.0	250.9	155.0 (55.4)	−29.1 (−10.4)
	15–17	320.2	419.2	266.5	99.0 (30.9)	−53.7 (−16.8)
2020–2021	5–9	68.8	68.8	75.8	0 (—)	7.0 (10.2)
	10–14	255.5	324.2	396.5	68.7 (26.9)	141 (55.2)
	15–17	290.6	316.9	359.4	26.3 (9.1)	68.8 (23.7)
2021–2022	5–9	55.1	84.1	102.4	29 (52.6)	47.3 (85.8)
	10–14	280.8	427.6	457.7	146.8 (52.3)	176.9 (63.0)
	15–17	309.7	402.8	395.9	93.1 (30.1)	86.2 (27.8)
2022–2023	5–9	61.2	80.2	81.5	19.0 (31.0)	20.3 (33.2)
	10–14	276.5	398.2	440.6	121.7 (44.0)	164.1 (59.3)
	15–17	310.5	385.2	394.1	74.7 (24.1)	83.6 (26.9)
**Cannabis-related disorders**						
2018–2019	5–9	1.7	2.4	3.0	0.7 (41.2)	1.3 (76.5)
	10–14	49.9	73.8	92.4	23.9 (47.9)	42.5 (85.2)
	15–17	290.2	366.6	417.2	76.4 (26.3)	127 (43.8)
2019–2020	5–9	3.3	4.0	5.4	0.7 (21.2)	2.1 (63.6)
	10–14	73.0	96.6	65.0	23.6 (32.3)	−8.0 (−11.0)
	15–17	331.0	405.8	319.4	74.8 (22.6)	−11.6 (−3.5)
2020–2021	5–9	8.1	7.6	13.9	−0.5 (−6.2)	5.8 (71.6)
	10–14	74.2	75.1	105.0	0.9 (1.2)	30.8 (41.5)
	15–17	341.7	337.1	389.9	−4.6 (−1.3)	48.2 (14.1)
2021–2022	5–9	12.9	6.9	14.3	−6.0 (−46.5)	1.4 (10.9)
	10–14	75.9	117.9	170.6	42.0 (55.3)	94.7 (124.8)
	15–17	318.6	398.9	488.5	80.3 (25.2)	169.9 (53.3)
2022–2023	5–9	13.4	12.8	18.6	−0.6 (−4.5)	5.2 (38.8)
	10–14	99.9	152.6	202.6	52.7 (52.8)	102.7 (102.8)
	15–17	367.6	431.4	500.1	63.8 (17.4)	132.5 (36.0)
**Lifestyle or life management factors**						
2018–2019	5–9	7.0	10.6	10.1	3.6 (51.4)	3.1 (44.3)
	10–14	69.3	117.6	131.1	48.3 (69.7)	61.8 (89.2)
	15–17	150.1	181.6	188.9	31.5 (21.0)	38.8 (25.8)
2019–2020	5–9	7.5	11.1	5.4	3.6 (48.0)	−2.1 (−28.0)
	10–14	83.3	129.5	86.8	46.2 (55.5)	3.5 (4.2)
	15–17	160.8	195.4	153.3	34.6 (21.5)	−7.5 (−4.7)
2020–2021	5–9	6.9	5.8	8.0	−1.1 (−15.9)	1.1 (15.9)
	10–14	94.7	124.1	172.7	29.4 (31.0)	78.0 (82.4)
	15–17	165.9	180.8	216.1	14.9 (9.0)	50.2 (30.3)
2021–2022	5–9	7.1	7.6	9.9	0.5 (7.0)	2.8 (39.4)
	10–14	112.6	164.1	175.6	51.5 (45.7)	63 (56.0)
	15–17	167.6	206.6	206.4	39.0 (23.3)	38.8 (23.2)
2022–2023	5–9	6.5	9.0	13.5	2.5 (38.5)	7.0 (107.7)
	10–14	104.0	137.4	183.8	33.4 (32.1)	79.8 (76.7)
	15–17	155.7	170.8	199.4	15.1 (9.7)	43.7 (28.1)
**Mood disorders**						
2018–2019	5–9	33.2	63.1	67.5	29.9 (90.1)	34.3 (103.3)
	10–14	124.2	192.2	222.9	68.0 (54.8)	98.7 (79.5)
	15–17	112.2	146.3	155.6	34.1 (30.4)	43.4 (38.7)
2019–2020	5–9	42.1	70.1	32.7	28.0 (66.5)	−9.4 (−22.3)
	10–14	145.4	230.1	139.0	84.7 (58.3)	−6.4 (−4.4)
	15–17	125.2	155.4	113.9	30.2 (24.1)	−11.3 (−9.0)
2020–2021	5–9	31.9	38.0	48.1	6.1 (19.1)	16.2 (50.8)
	10–14	147.5	169.2	216.4	21.7 (14.7)	68.9 (46.7)
	15–17	124.5	134.9	164.4	10.4 (8.4)	39.9 (32.0)
2021–2022	5–9	33.0	49.4	54.5	16.4 (49.7)	21.5 (65.2)
	10–14	168.5	234.1	261.8	65.6 (38.9)	93.3 (55.4)
	15–17	136.1	183.7	193.0	47.6 (35.0)	56.9 (41.8)
2022–2023	5–9	36.2	44.1	52.1	7.9 (21.8)	15.9 (43.9)
	10–14	187.4	243.2	285.5	55.8 (29.8)	98.1 (52.3)
	15–17	157.1	187.1	207.4	30.0 (19.1)	50.3 (32.0)
**Poisoning by drugs, initial encounter**						
2018–2019	5–9	55.9	50.1	56.6	−5.8 (−10.4)	0.7 (1.3)
	10–14	167.0	235.6	267.5	68.6 (41.1)	100.5 (60.2)
	15–17	318.3	434.9	431.2	116.6 (36.6)	112.9 (35.5)
2019–2020	5–9	55.8	53.9	41.1	−1.9 (−3.4)	−14.7 (−26.3)
	10–14	195.4	250.8	219.6	55.4 (28.4)	24.2 (12.4)
	15–17	347.2	445.3	370.6	98.1 (28.3)	23.4 (6.7)
2020–2021	5–9	56.5	46.9	57.7	−9.6 (−17.0)	1.2 (2.1)
	10–14	231.5	324.7	408.7	93.2 (40.3)	177.2 (76.5)
	15–17	381.8	460.0	519.3	78.2 (20.5)	137.5 (36.0)
2021–2022	5–9	61.6	50.6	66.4	−11.0 (−17.9)	4.8 (7.8)
	10–14	301.3	386.1	371.5	84.8 (28.1)	70.2 (23.3)
	15–17	413.6	532.2	506.9	118.6 (28.7)	93.3 (22.6)
2022–2023	5–9	66.2	61.2	72.6	−5.0 (−7.6)	6.4 (9.7)
	10–14	264.9	319.3	358.1	54.4 (20.5)	93.2 (35.2)
	15–17	397.1	472.4	481.2	75.3 (19.0)	84.1 (21.2)
**Symptoms of mental and substance use conditions**						
2018–2019	5–9	152.8	255.2	299.6	102.4 (67.0)	146.8 (96.1)
	10–14	343.5	563.6	671.4	220.1 (64.1)	327.9 (95.5)
	15–17	289.8	394.1	441.9	104.3 (36.0)	152.1 (52.5)
2019–2020	5–9	194.5	306.2	141.4	111.7 (57.4)	−53.1 (−27.3)
	10–14	470.1	675.6	382.9	205.5 (43.7)	−87.2 (−18.5)
	15–17	384.6	465.5	346.3	80.9 (21.0)	−38.3 (−10.0)
2020–2021	5–9	148.4	184.7	224.4	36.3 (24.5)	76.0 (51.2)
	10–14	420.2	556.9	700.4	136.7 (32.5)	280.2 (66.7)
	15–17	393.3	438.2	496.5	44.9 (11.4)	103.2 (26.2)
2021–2022	5–9	175.7	240.2	284.4	64.5 (36.7)	108.7 (61.9)
	10–14	532.5	732.7	842.7	200.2 (37.6)	310.2 (58.3)
	15–17	433.2	537.9	595.7	104.7 (24.2)	162.5 (37.5)
2022–2023	5–9	198.5	251.1	282.5	52.6 (26.5)	84.0 (42.3)
	10–14	560.2	734.2	835.8	174.0 (31.1)	275.6 (49.2)
	15–17	477.5	556.9	623.5	79.4 (16.6)	146.0 (30.6)

* To reduce artifactual impact from changes in reporting patterns, analyses were restricted to facilities with a coefficient of variation of emergency department visits ≤40 and average weekly informative discharge diagnosis ≥75% complete with consistent discharge diagnosis code formatting during January 2018–June 2023.

^†^ Percent change was calculated as visits during the fall school semester (calendar weeks 37–53, September–December) and spring school semester (calendar weeks 1–23, January–June) separately compared with visits during the summer period (calendar weeks 24–36, June–September).

^§^ National Syndromic Surveillance Program is a collaboration among CDC; local and state health departments; and federal, academic, and private sector partners. https://www.cdc.gov/nssp/index.html

## Discussion

Each year, during 2018–2023, the number and proportion of weekly ED visits for eight mental and behavioral health conditions displayed seasonal increases during the fall and spring school semesters compared with the summer period; timing of increase varied by specific conditions. Trends suggest that students might need additional mental health support during the back-to-school period in the fall and throughout the academic year.

Visit patterns during the 2020 spring school semester showed a relative increase in incidence (visit ratio >1) and lower mean weekly visit counts (percent change <0) compared with the 2019 summer period. These findings indicate that the relative proportion of visits was higher while the mean weekly number of visits was lower and was likely influenced by the public health emergency declaration for the COVID-19 pandemic in March 2020 (*1**,**3*). 

These findings raise concerns about the challenges U.S. children and adolescents face in the school environment (*4*). Several factors might contribute to these increases. Children and adolescents can experience unique school-related stressors,** including transitioning into the school year or attending a new school, academic performance pressure and testing, and in-school bullying and peer victimization. Social anxiety might lead to worsening mental health, resulting in a visit to an ED (*5**–**7*). School- and provider-based screenings and assessments for mental health usually increase at the start of the school year, prompting referral for care (*8*). Mental and behavioral health conditions might be recognized by school staff members when they manifest in classroom behavioral issues (e.g., disruption in class, poor attendance, and poor academic performance), or when students disclose mental health challenges.

Engaging children and adolescents in social and emotional learning (SEL) programs can promote their emotional well-being. School-based SEL programs^††^ provide students and teachers with tools to cope with stressors. Other strategies that have been shown to be effective at promoting and maintaining emotional well-being among children and adolescents include pediatric mental health care access programs; suicide prevention gatekeeper trainings; trauma and grief interventions; crisis intervention and response services; peer-led approaches to encourage students to seek help; evidence-based comprehensive school health–education curriculum that includes lessons on mental health disorders, self-care, substance use prevention and sexual health education, providing access to local and national mobile crisis services, and expanding community-based service alternatives (*2**,**9**,**10*).

Multisector collaboration and coordination, including government, education, and community organizations, are needed to promote and prioritize child and adolescent mental health and to avoid placing the responsibility of improvement solely on educational institutions.^§§^ Evidence-based strategies (e.g., CDC’s Preventing Adverse Childhood Experiences [ACEs]: Leveraging the Best Available Evidence resource)^¶¶^ offer options for a comprehensive and systems-level approach to supporting children and families. State and local government agencies and school partners can collaborate when addressing the behavioral health of children. CDC approaches, including the Whole School, Whole Community, Whole Child model,*** What Works in Schools program,^†††^ Suicide Prevention Resource for Action,^§§§^ and ACEs training module can be useful for schools seeking to support or enhance protective factors and respond using trauma-informed methods (*7**,**9*). Government agencies can collaborate to establish tailored and culturally responsive messaging^¶¶¶^^,^****^,^^††††^ for various audiences (e.g., parents and caregivers, students, community leaders, health care providers, and educational professionals), including social media campaigns about students’ mental health needs during certain times of the year.^§§§§^

### Limitations

The findings in this report are subject to at least five limitations. First, NSSP ED visit data are a convenience sample and are not nationally representative. Second, ED visits represent unique events, not individual persons, and might reflect multiple visits for one person. Third, HCUP Clinical Classifications Software Refined categories are not mutually exclusive; codes can appear in more than one category. Fourth, results for children aged 5–9 years should be interpreted with caution, particularly data about suicidal ideation or self-harm, because of low visit volume and uncertainty about intentionality. Finally, because school start and end dates vary within and across regions, some ED visits might be misclassified, resulting in underestimation of the extent of the increase in number of ED visits for mental and behavioral health conditions; many such visits can occur outside of EDs and reasons for changes in ED visit patterns cannot be ascertained from these data.

### Public Health Implications

Systemic changes that prioritize protective factors (e.g., physical activity; nutrition; sleep; social, community, or faith-based support; and inclusive school and community environments) and well-being promotion might improve mental health among children and adolescents long before a trip to an ED is needed. These changes include consideration of the seasonal timing of increases in child and adolescent mental and behavioral health conditions; efforts to incorporate preparedness for mental health concerns into programmatic planning, especially during back-to-school; prevention of conditions that increase risk for mental disorders; early identification of mental health disorders; and targeted interventions. Parents and caregivers, educators, health care providers, and others who regularly interact with children and adolescents can learn about signs and symptoms of mental distress^¶¶¶¶^ and monitor children and adolescents for possible increases in mental distress in the weeks leading up to and during the academic year.

## Footnotes

* https://www.cdc.gov/childrensmentalhealth/features/COVID-19-helping-children-transition-back-to-school.html; https://www.cdc.gov/healthyyouth/protective/school_connectedness.htm

† To reduce artifactual impact from changes in reporting patterns, analyses were restricted to facilities with a coefficient of variation for ED visits ≤40 and average weekly informative discharge diagnosis ≥75% complete with discharge diagnosis code formatting during January 2018–June 2023. https://www.cdc.gov/nssp/index.html

§ ED visits with multiple codes could be counted across more than one category; however, if multiple codes in a single visit mapped to the same category, the visit was counted only once; a full list of categories and corresponding codes is available at the HCUP website. https://www.hcup-us.ahrq.gov/toolssoftware/ccsr/dxccsr.jsp

¶ 45 C.F.R. part 46.102(l)(2), 21 C.F.R. part 56; 42 U.S.C. Sect. 241(d); 5 U.S.C. Sect. 552a; 44 U.S.C. Sect. 3501 et seq.

**
https://www.scientificamerican.com/article/childrens-risk-of-suicide-increases-on-school-days/

††
https://www.apa.org/monitor/2020/09/classroom-connections

§§
https://www.hhs.gov/sites/default/files/surgeon-general-youth-mental-health-advisory.pdf

¶¶ https://www.cdc.gov/violenceprevention/pdf/ACEs-Prevention-Resource_508.pdf

***
https://www.cdc.gov/healthyschools/wscc/index.htm

†††
https://www.cdc.gov/healthyyouth/whatworks/index.htm

§§§
https://www.cdc.gov/suicide/resources/prevention.html

¶¶¶
https://www2.ed.gov/documents/students/supporting-child-student-social-emotional-behavioral-mental-health.pdf

****
https://store.samhsa.gov/product/Identifying-Mental-Health-and-Substance-Use-Problems-of-Children-and-Adolescents-A-Guide-for-Child-Serving-Organizations/SMA12-4700

††††
https://vetoviolence.cdc.gov/apps/aces-training/#/edu#top

§§§§
https://knowledgerepository.syndromicsurveillance.org/mental-and-behavioral-health-resources

¶¶¶¶
https://www.cdc.gov/childrensmentalhealth/basics.html

## References

[1] Anderson KN, Johns D, Holland KM, Emergency department visits involving mental health conditions, suicide-related behaviors, and drug overdoses among adolescents—United States, January 2019–February 2023. MMWR Morb Mortal Wkly Rep 2023;72:502–12. 10.15585/mmwr.mm7219a1 37167103 PMC10208370

[2] Bitsko RH, Claussen AH, Lichstein J, ; Mental health surveillance among children—United States, 2013–2019. MMWR Suppl 2022;71(Suppl-2):1–42. 10.15585/mmwr.su7102a1 35202359 PMC8890771

[3] Radhakrishnan L, Carey K, Hartnett KP, Pediatric emergency department visits before and during the COVID-19 pandemic—United States, January 2019–January 2022. MMWR Morb Mortal Wkly Rep 2022;71:313–8. 10.15585/mmwr.mm7108e1 35202351

[4] Stanley IH, Horowitz LM, Bridge JA, Wharff EA, Pao M, Teach SJ. Bullying and suicide risk among pediatric emergency department patients. Pediatr Emerg Care 2016;32:347–51. 10.1097/PEC.0000000000000537 26417959 PMC4808508

[5] Carbone JT, Holzer KJ, Vaughn MG. Child and adolescent suicidal ideation and suicide attempts: evidence from the healthcare cost and utilization project. J Pediatr 2019;206:225–31. 10.1016/j.jpeds.2018.10.017 30413313

[6] Copeland JN, Babyak M, Inscoe AB, Maslow GR. Seasonality of pediatric mental health emergency department visits, school, and COVID-19. Pediatr Emerg Care 2022;38:e1673–7. 10.1097/PEC.0000000000002671 35319855 PMC9722329

[7] Lueck C, Kearl L, Lam CN, Claudius I. Do emergency pediatric psychiatric visits for danger to self or others correspond to times of school attendance? Am J Emerg Med 2015;33:682–4. 10.1016/j.ajem.2015.02.055 25797865

[8] Goldstein AB, Silverman MA, Phillips S, Lichenstein R. Mental health visits in a pediatric emergency department and their relationship to the school calendar. Pediatr Emerg Care 2005;21:653–7. 10.1097/01.pec.0000181420.56729.4f 16215467

[9] Foster CE, Horwitz A, Thomas A, Connectedness to family, school, peers, and community in socially vulnerable adolescents. Child Youth Serv Rev 2017;81:321–31. 10.1016/j.childyouth.2017.08.011 30202142 PMC6128354

[10] Kim WJ; American Academy of Child and Adolescent Psychiatry Task Force on Workforce Needs. Child and adolescent psychiatry workforce: a critical shortage and national challenge. Acad Psychiatry 2003;27:277–82. 10.1176/appi.ap.27.4.277 14754851

